# Acute toxicity of water and aqueous extract of soils from Champotón river in *Lactuca sativa L.*

**DOI:** 10.1016/j.toxrep.2018.05.009

**Published:** 2018-05-17

**Authors:** Carlos A. Chan-Keb, Claudia M. Agraz-Hernández, Roman A. Perez-Balan, Mónica I. Gómez-Solano, Teresita D.N.J. Maldonado-Montiel, Baldemar Ake-Canche, Eduardo J. Gutiérrez-Alcántara

**Affiliations:** a Facultad de Ciencias Químico Biológicas, Universidad Autónoma de Campeche, Av. Agustín Melgar s/n entre Juan de la Barrera y Calle 20, Col. Buenavista, A.P. 24039, San Francisco de Campeche, Campeche, Mexico; b Instituto EPOMEX, Universidad Autónoma de Campeche, Av. Agustín Melgar s/n entre Juan de la Barrera y Calle 20, Col. Buenavista, A.P. 24039, San Francisco de Campeche, Campeche, Mexico

**Keywords:** Bioassay, *Lactuca sativa*, Champotón river, Phytotoxicity, Soil–water solution, Environmental toxicology

## Abstract

The seeds germination, stem and root elongation of water lettuces (*Lactuca sativa L*) were used to evaluate the phytotoxicity of water and soil of Champotón River, Campeche, Mexico. water and soil sample were collected from two sampling sites. Lettuce seeds were exposed to three different dilutions of water and aqueous extract of soil. Significant differences on germination, root and stem elongation of lettuce were detected. Water showed more phytotoxic effect than aqueous extract of soil and inhibitory concentration 50 (IC_50_) for radicle exposed to river water was 52% while stem elongation was 69%. Due to paucity the previously available phytotoxicological studies onto Champotón River, it is now essential to allocate time and resources to consider development of suitable chronic phytotoxicity tests.

## Highlights

• Inhibitory effect on elongation for radicle and stem of *Lactuca sativa* was detected.
• Champotón river water showed more phytotoxic effect than aqueous extracts of the soil.
• Radicula and stem of lettuces showed variations in growth when exposed to different water dilutions from Champoton river.

## Graphical abstract

**Figure:**
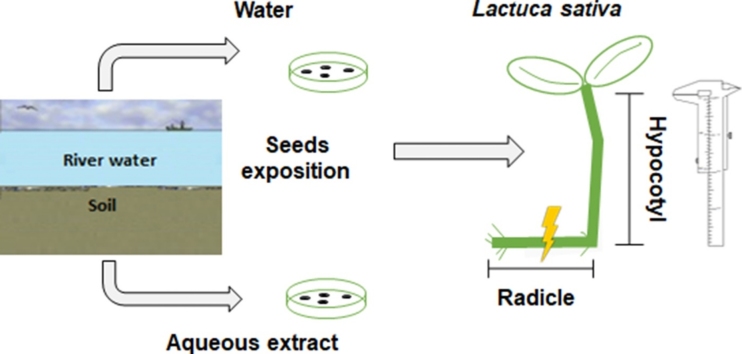

## 1 Introduction

Bioassay toxicity *Lactuca sativa* seeds is a 120 h of exposure static acute toxicity test with, which allows evaluating the phytotoxic effects of pure compounds or complex mixtures in the process of seed germination and development of seedlings in the first days of growth [1]. The success or aptitude of a seedling to establish itself in a certain environment is of great importance to guarantee the survival of the species. The evaluation of the development of the radicle and the hypocotyl constitute representative indicators to determine the capacity of establishment and development of the plant [2]. Using bioassays with plants represents a quick and economical method for the characterization of the toxicity of environmental samples [3].

Champotón River, the main surface stream of the Yucatan Peninsula is located in Southeastern Mexico on an area with high content of karstic material. This zone is classified as a priority hydrological region by *Comisión Nacional para el Conocimiento y Uso de la Biodiversidad* since 2002 [4]. This river is within the so-called hot-spot of Mesoamerica, whose main problems are agricultural waste input, discharges from sugar mills and contamination by domestic sewage at the river mouth [5]. Champotón river is particularly relevant due to the attributes associated with ecosystems susceptible to conservation, although it faces great challenges due to deforestation and insufficient research on toxic effects of pollution from non-point sources [6].

Thus, this study is to determine acute toxic effects of exposition to water and aqueous extract of soils of Champotón River in germination and elongation of radicle and hypocotyl of lettuces (*Lactuca sativa L*).

## 2 Material and methods

**Fig. 1:**
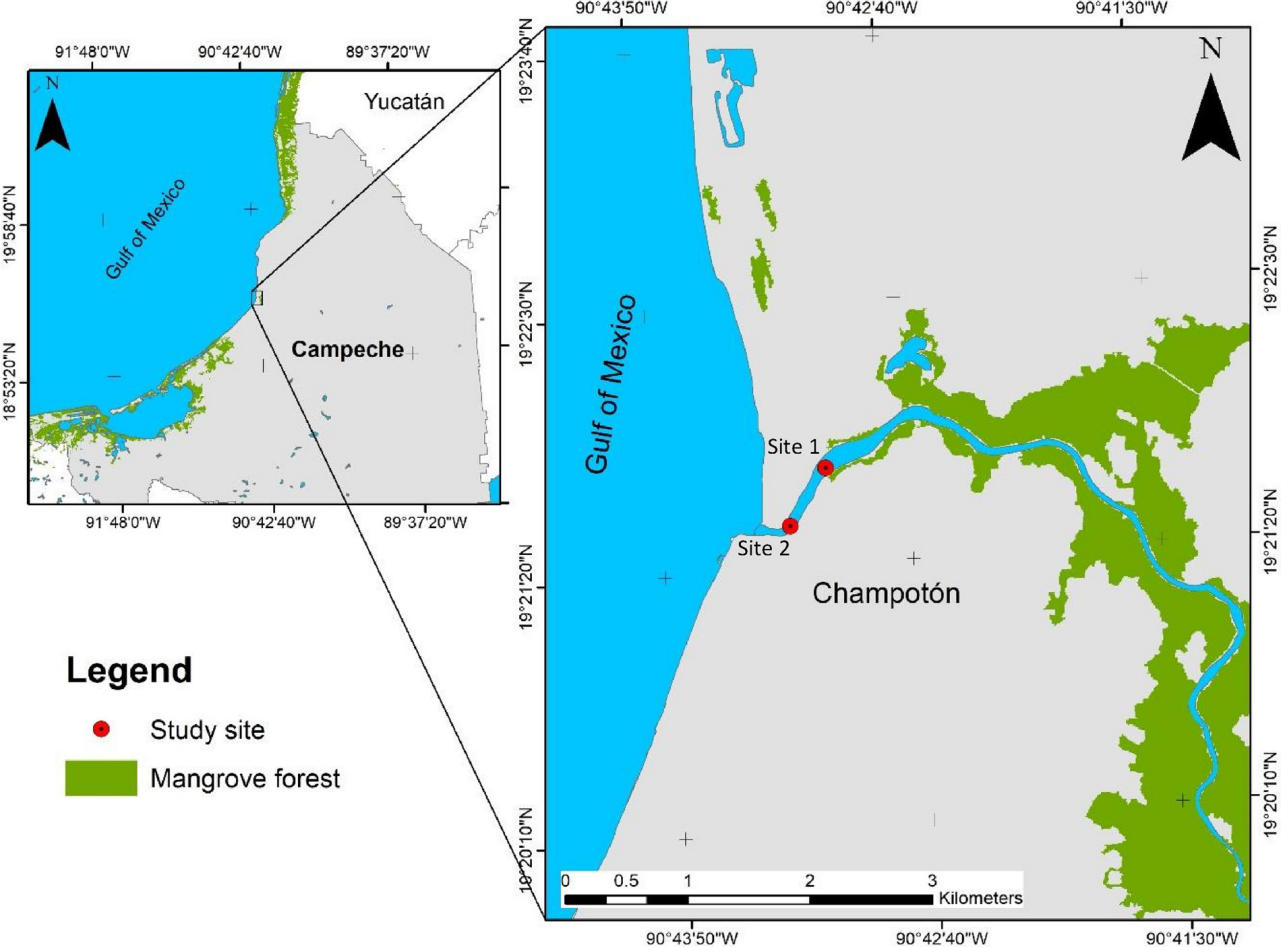
Geographic location of the Champotón River in the state of Campeche, Mexico and location of the sampling sites.

The study area was the Champotón River which empties into the Gulf of Mexico and gives its name to the municipal capital of Champotón. The river borders the Gulf of Mexico and the municipality of Campeche to the north; to the east with the municipalities of Campeche, Hopelchén, Calakmul and Escárcega; to the south with the municipalities of Escárcega and Carmen and to the west with the municipality of Carmen and the Gulf of Mexico (Fig. 1). The Champotón River is located in the humid subtropics of southeastern Mexico in terrain with high content of karstic material, is the main surface stream in the Yucatán Peninsula. This River encompasses a basin of nearly 650 km^2^ with a mean volume of 483.93 million m^3^/year and a longitude of 48 km. Its mean discharge volume is estimated at 0.2 × 10^9^ m^3^/year [7]. The breadth of the mouth is close to 85 m because almost the entire basin is nearly at sea level; the mean depth of the river ranges from 2.5 to 4.5 m, depending on the tides. The river is fed by underground rivers including the “Desempeño” and “Las Pozas”. In the catchment area, several types of vegetation have been identified, including mangrove, tropical rainforest, subperennifolium rainforest, flooded lowland forest, flooded palm, thorn scrub, savanna and cultivated grassland. The Champotón River suffers human impacts from towns like San Juan Carpizo, Canasayab, Pabox and Champotón. The main human activities in the river basin are agriculture, in addition to fishing and tourism. However, a burn cane and sugar-mill affects almost the totality of the basin [8].

The samples’ collection was carried out in July 2017, two sampling sites were chosen along the Champotón River, the first located one km upstream entering the urban area and the second sampling site was located where the river stream across the urban zone, at 3 km downstream from first sampling site. For each sampling site, a water sample of 500 mL at 10 cm depth was collected by immersing a polypropylene bottle previously washed and rinsed with distilled water, also for each sampling site, a sample of approximate 300 g of soil were collected by using a borer PVC of two inches of diameter. The samples’ collection was carried out upstream one after the other in the same day. In situ measurements of pH, temperature, redox and salinity were made in triplicate for each sample using a HACH HQ40D Portable Multimeter equipment and a refractometer A&O with measurement range of 0–100 PSU (practical units of salinity) [9]. For acute toxicity test the water extract of the soils was used, obtained after 2 h of shaking, and extraction with distilled water.

For acute toxicity determination 3 treatments per sample at 100, 50 and 25% of initial solution concentration for water and water-extract of soils with duplicate and distilled water as growth control (negative) for a total of 30 experimental units were made. For each unit, 25 seeds of *Lactuca sativa* (Vita-Los Molinos) were placed in a 90 mm diameter polyethylene container, with filter paper (Whatman^®^ No. 3) in the bottom as the support. Afterward, 10 mL of the different solution concentration were applied. All units were kept in controlled ambient temperature of 24 °C ± 1 for 120 h according to EPA Test Guidelines for phytotoxicity [10]. Response variables analyzed were seed germination, root and stem elongation and inhibitory concentration 50 (IC_50_) by PROBIT analysis. Radicle and hypocotyl elongation inhibition was calculated by the equation: percentage inhibition (%) = 100 − ((M*100)/C) where M is the average elongation per treatment and C is the average elongation for negative control (distillate water) [11].

To evaluate the toxicity effect of the water and soil of the Champotón River, the morphometric variables (seed germination, radicle and hypocotyl elongation) of *Lactuca sativa* were compared between different treatments, using one-way ANOVA. Subsequent post hoc analysis Fisher’s Least Significant Difference test (LSD) was applied to determine significant differences among means at p ≤ 0.05 level of significance. The normality was analyzed by the Shapiro-Wilk test, using p ≤ 0.05 significance level, data not displaying a normal distribution were square root transformed. Also, PROBIT analysis was applied to determine the inhibitory concentration (IC_50_). All statistical were performed using STATISTICA V.12 (^©^ Copyright StatSoft, Inc., 1984–2014).

## 3 Results

Physicochemical measure of the water and soil samples undiluted (100%) are shown in Table 1. For sampling site 1, water sample was labeled “1 W” and soil sample as “1S”, while water and soil of sampling site 2 were labeled “2 W” and “2S” respectively. There were 4 samples in total. For site 1, the soil was silty-sandy type while the soil for site 2 was sandy.

**Table 1:** Physicochemical characteristics of the water and soil of the Champotón River used for the bioassay with *Lactuca sativa*, For mean values ± standard deviation the measurement was taken three times for each sample type.

Sampling site	Sample type	pH	Temperature (°C)	Redox potential (mV)	Practical Salinity Unit (PSU)
1	water (1 W) n = 1	6.61 ± 0.05	26.0 ± 0.05	10.1 ± 4	13 ± 3
	Soil (1S) n = 1	7.6 ± 0.02	25.1 ± 0.08	−16.5 ± 5	24 ± 1
2	Water (2 W) n = 1	6.95 ± 0.01	28.0 ± 0.03	11.5 ± 7	12 ± 2
	Soil (2S) n = 1	7.8 ± 0.03	26.5 ± 0.09	−17.5 ± 6	27 ± 1

**Fig. 2:**
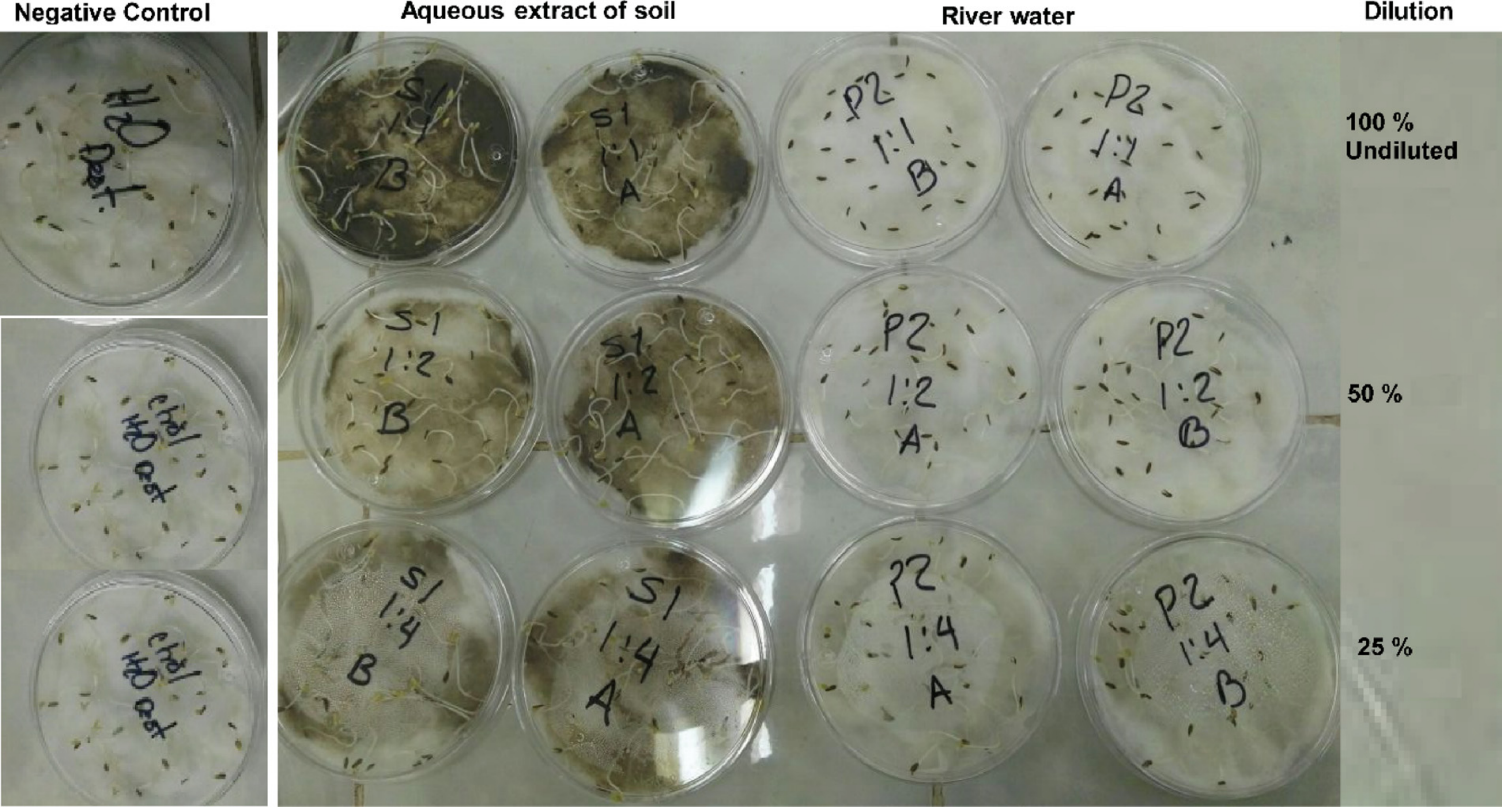
Germination and elongation alterations observed on seeds of Lactuca sativa exposed to different treatments.

**Fig. 3:**
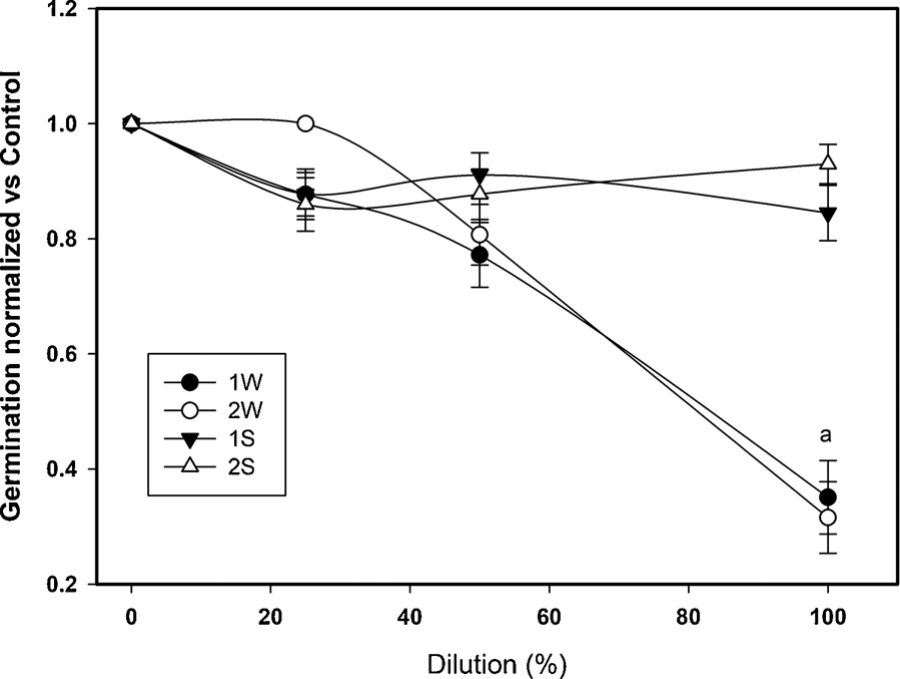
Effect of exposure to water and soil of the Champotón river on the germination of lettuce seeds. Number in label indicates site and letter indicates type of sample “W” for water and “S” for Soil. Error bars represent standard deviation, a = significantly different.

Fig. 2 shows some examples of the bioassay results with lettuce seeds of this study, for the column marked as “Negative Control” seedlings have germinated and grown. For the column “aqueous extract of soil”, effect on the germination and growth for lettuce seeds exposed to different dilutions of aqueous extract soil are presented in duplicate, in this column seed growth was shown in all trials. The Petri dishes containing bioassays of lettuce seeds exposed to the different dilutions of river water for sampling site 2 (labeled as P2) are also shown in Fig. 2. The seeds exposed to the 100% of water displayed few germinations while the seeds exposed to 25% dilution developed seedlings. Fig. 3 shows the normalized data of the germination percentage of the lettuce seeds with respect to distilled water negative control. The seeds germinated in the negative control was considered 100% germination. Here, only samples of 100% river water, from both sampling sites, cause approximately 60% decrease in germination and this value is statistically significant with respect to the rest of the dilutions, while aqueous sediment extracts did not shown significant differences in germination. The inhibitory concentration 50 (IC_50_) in seeds germination was 63% water dilution water from both sampling sites.

**Fig. 4:**
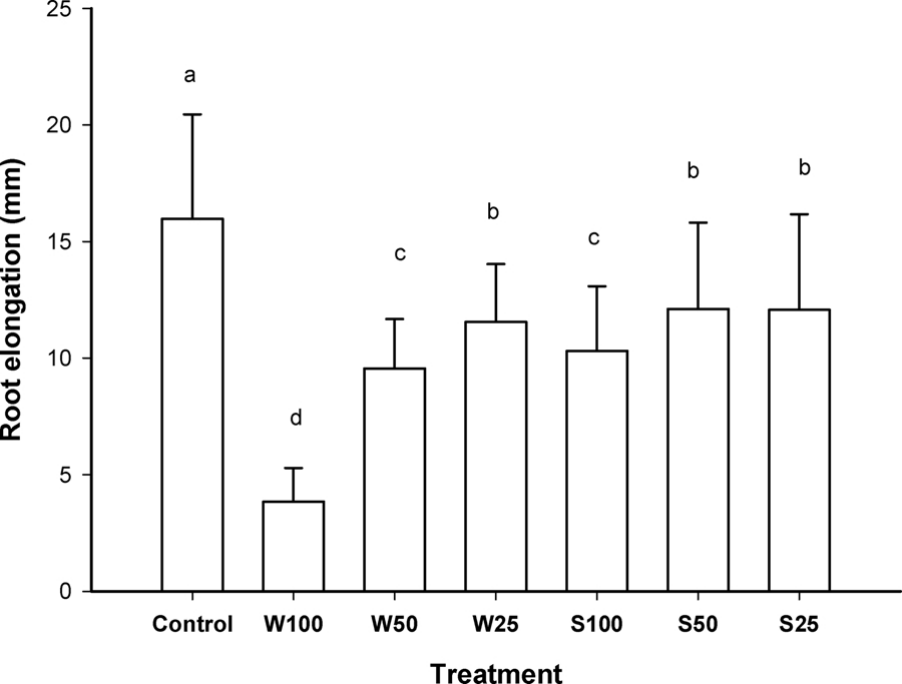
Radicle elongation of lettuces versus concentration of water and aqueous extracts of soil. Treatments that do not share a letter are significantly different. Error bars represent standard deviation.

**Fig. 5:**
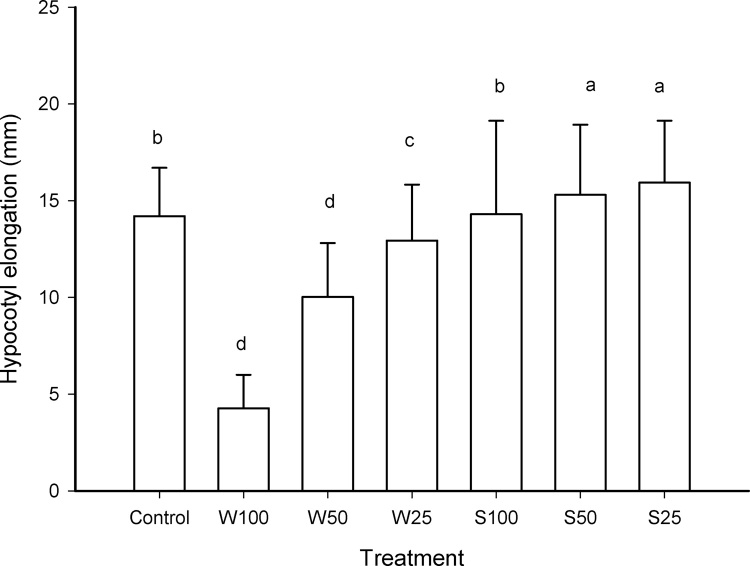
Hypocotyl elongation of lettuces versus concentration of water and aqueous extracts of soil. Treatments that do not share a letter are significantly different. Error bars represent standard deviation.

Exposure to different concentrations of water from the Champotón River also caused a decrease in elongation in the radicle and hypocotyl. However, the aqueous extracts of soil did not cause a decrease in elongation stems and roots of the lettuces. There was no significant difference between sampling sites. From the above, considering all the samples from the same site, decreased elongation of radicles with respect to the control was observed, being the treatment with water at 100% (W100) that presented the greatest effect (Fig. 4). Hypocotyls elongation decreased with water treatments, but not in treatments with soils aqueous extracts in contrast an increase in stem elongation was observed for the exposure of 50 and 25% dilutions (S50 and S25). Exposure to the soil extract at 100% (S100) did not show significant difference with respect to the negative control (Fig. 5). The inhibitory concentration 50 for the root exposed to water was 52%, while the effect of the IC_50_ of the water in the hypocotyl was 69%. However, for both stems and roots the IC_50_ could not be determined for aqueous extracts of the sediment because the higher concentration did not produce inhibition of the elongation.

## 4 Discussion

Germination and elongation could be due to water-soluble contaminants capable of inhibiting the growth of the root and stem of lettuce. Similar finding has been reported by other authors who also tested the effects of exposure to water and sediments of rivers polluted with metals for example Biruk and collaborators [12] describe toxic effects of 5 different types of sediment extracts from Matanzas-Riachuelo basin (Argentina), 2 aqueous extracts and three organic extracts. They found lettuce seeds were more sensitive to extracts that contained inorganic elements than organic extracts. Pollutants in the waters used in this study is possible. Some research have reported heavy metals, persistent organic compound residues (POCs) including polychlorinated biphenyls (PCBs), Hexachlorocyclohexanes (HCH), Aldrin-related pesticides, heptachlor, DDT and Polycyclic Aromatic hydrocarbons (PAH) in the water column of different points in Champotón River [13,14]. In 2016 Dzul-Caamal and collaborates [8] have reported high concentrations of metals like Cu, Fe, Mn, Pb and Zn in river waters, lettuce seeds are extremely sensitive to metals [15], maybe these metals are main causes for inhibition effects detected in our study. In addition, other pollutants including organic matter and nitrogen could be involved in the observed effects [16].

Regarding to the possible effect of salinity, in agreement with Al-Maskri and collaborates [17], it seems the level of salinity detected in this study does not influence the decrease germination and the elongation of the stem and root of lettuces.

In general terms, for the germination and elongation for roots of lettuce, the exposure to the water of the Champotón River produced more inhibitory effects than the exposure to the aqueous soil extract. This effect observed by exposure to water may be due to the bioavailability of the agents that cause the inhibition [18,19]. If the inhibitors were solubilize in water, they would become more bioavailable than if they were adsorbed to the sediment particles [20]. Although the lettuce seeds were exposed to aqueous extracts of sediments, perhaps the absence of inhibitory effect was due to the inhibitory agents might not be extracted with the distilled water [21].

Trujillo and collaborators [6] indicated the Champotón river water is not fit for use in the public water supply system and is marginally fit for sensitive fish. The Champotón River is not affected by pollution from industries and large cities; the major sources of pollution are non-point sources from agriculture, livestock-related chemical residues, and the input of organic matter from small human settlements near the river.

From the above, more phytotoxicity studies are needed, as well as the quantification of pollutants like heavy metals, nitrogen and organic matter in water and sediments of Champotón River that could help identify the phytotoxic origin in *Lactuca sativa* model.

Although our study did not include the monitoring of contaminants, this work approach allowed the detection of stress in the germination and elongation of lettuce seeds caused by water exposure of the Champotón River. Inhibition of elongation responses of inhibition provided evidence that the health status of the Champotón River-associated flora may be compromised with natural and anthropogenic stressors. However, we lack of previous studies of the Champotón River that can support the hypothesis of the Phytotoxic effect. Therefore, our study is the first to use acute phytotoxicity to assess the environmental conditions of the Champotón River, providing valuable background data for future studies.

## 5 Conclusions

This study describes phytotoxic effect by water and sediments of Champotón River for first time. The phytotoxicity was related with inhibitory effects on the radicle, hypocotyl elongation and germination of *Lactuca sativa* seeds. Thus, the exposition to water of Champotón River, exerted a stronger effect on seed germination and root elongation in lettuce. The test employed enables the identification of different levels of phytotoxicity in the water and soils samples further, the bioassay with *Lactuca sativa* proved to be efficient, sensitive, inexpensive, quick and reproducible.

## Transparency document

The Transparency document associated with this article can be found in the online version.

Transparency document
